# Role of the N-terminus for the stability of an amyloid-β fibril with three-fold symmetry

**DOI:** 10.1371/journal.pone.0186347

**Published:** 2017-10-12

**Authors:** Christian A. Söldner, Heinrich Sticht, Anselm H. C. Horn

**Affiliations:** Bioinformatik, Institut für Biochemie, Emil-Fischer-Centrum, Friedrich-Alexander-Universität Erlangen-Nürnberg (FAU), Erlangen, Germany; Torrey Pines Institute for Molecular Studies, UNITED STATES

## Abstract

A key player in Alzheimer’s disease is the peptide amyloid-beta (Aβ), whose aggregation into small soluble oligomers, protofilaments, and fibrils finally leads to plaque deposits in human brains. The aggregation behavior of Aβ is strongly modulated by the nature and composition of the peptide’s environment and by its primary sequence properties. The N-terminal residues of Aβ play an important role, because they are known to change the peptide’s aggregation propensity. Since these residues are for the first time completely resolved at the molecular level in a three-fold symmetric fibril structure derived from a patient, we chose that system as template for a systematic investigation of the influence of the N-terminus upon structural stability. Using atomistic molecular dynamics simulations, we examined several fibrillar systems comprising three, six, twelve and an infinite number of layers, both with and without the first eight residues. First, we found that three layers are not sufficient to stabilize the respective Aβ topology. Second, we observed a clear stabilizing effect of the N-terminal residues upon the overall fibril fold: truncated Aβ systems were less stable than their full-length counterparts. The N-terminal residues Arg5, Asp7, and Ser8 were found to form important interfilament contacts stabilizing the overall fibril structure of three-fold symmetry. Finally, similar structural rearrangements of the truncated Aβ species in different simulations prompted us to suggest a potential mechanism involved in the formation of amyloid fibrils with three-fold symmetry.

## Introduction

Alzheimer's Disease (AD) has become the most prevalent neurodegenerative disorder in developed countries [1, 2]. Hallmark of AD is the aggregation of amyloid-β (Aβ) monomers to oligomers, fibrils and finally plaques as explained by the Aβ cascade hypothesis [3–5]. From extensive experimental work it is known, that Aβ peptide's aggregation behavior is influenced by many external and internal factors like pH-value, temperature, ionic strength, peptide length, or point mutations [6–12].

A particular role for the aggregation behavior plays the N-terminus of Aβ (residues 1–8). Evidence for the importance of the N-terminal region in Aβ aggregation arises from the fact that familial mutations occur in the N-terminus, e.g. the English (His6Arg) and the Tottori (Asp7His) mutant, which show accelerated fibril formation [13, 14]. Phosphorylation of Ser8 significantly changes the fibril topology [15], whereas a Ser8Cys mutation favors the formation of soluble dimers by oxidative crosslinking [16, 17]. Metal ions like Zn^2+^ bind to N-terminal residues and accelerate amyloid formation [18–20]. On the other hand, effective antibodies against the N-terminal region of Aβ inhibit fibrillogenesis [21]. A comparison of full-length Aβ to its N-terminally truncated or pyroglutamate containing variants with regards to the aggregation behavior also points towards the particular role of the N-terminus: truncated species, which are found in brain deposits as well as in serum and cerebrospinal fluid[22–26], possess a distinct aggregation behavior with a possibly altered aggregation pathway [27]. Despite this demonstrated importance of the N-terminus, most experimental investigations of fibrillar Aβ structures revealed disordered N-termini in the fibril state [28–31]. Based on this observation, it was speculated that the N-terminus rather plays a role for the aggregation process itself than for the stability of the mature fibril.

**Fig 1:**
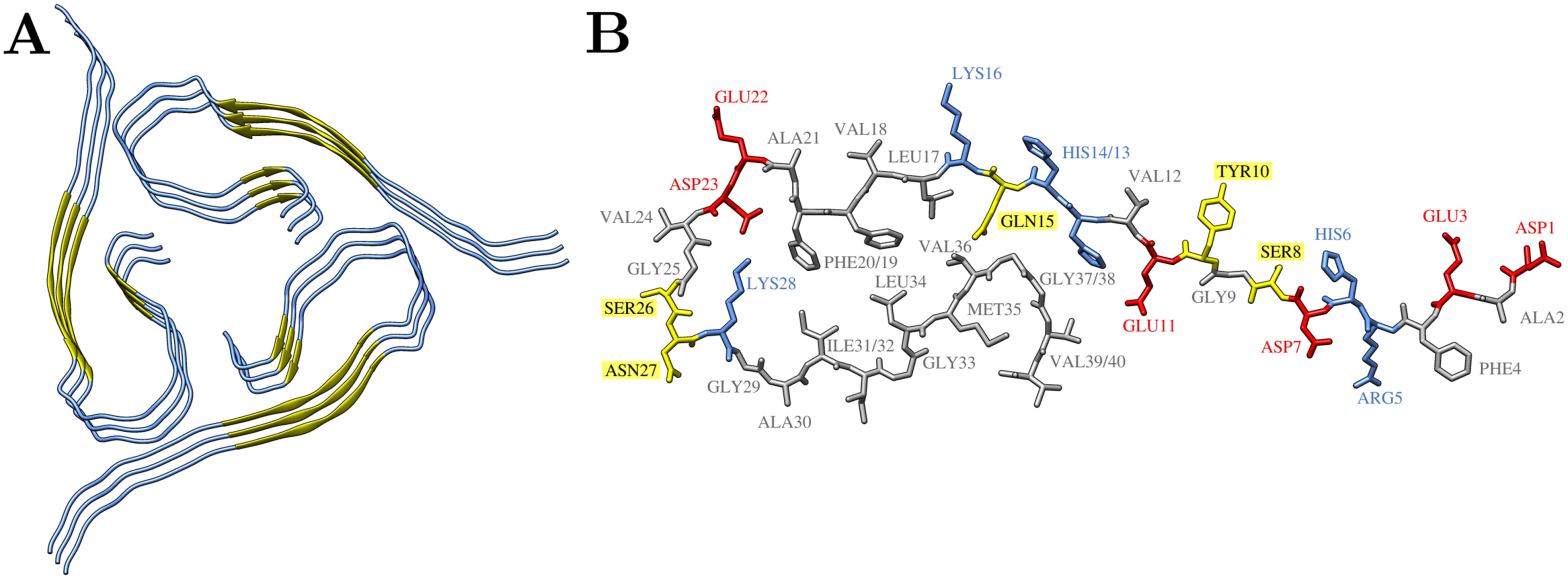
Overview of the three-fold symmetric fibril structure of Aβ1–40 (PDB-code 2M4J, Model 1, [32]). (A) Three fibril layers of full-length Aβ40 in cartoon representation. The N-terminal and C-terminal β-sheets are shown in yellow arrows. (B) Single Aβ40 peptide chain with sequence D_1_AEFRHDSGY_10_EVHHQKLVFF_20_AEDVGSNKGA_30_IIGLMVGGVV_40_ in sticks representation; residues are colored according to their properties: basic–blue, acidic–red, polar–yellow, hydrophobic–grey. Hydrogen atoms are omitted for clarity.

One rare example for a mature fibril with a structured N-terminus comes from a solid state NMR structure of a wild-type Aβ40 fibril isolated from an AD patient (PDB code 2M4J; cf Fig 1)[32]. The structure shows a three-fold symmetry around the fibril axis with a central water pore. The three filaments composing the fibril consist of Aβ monomers in an overall strand-loop-strand conformation (cf Fig 1B), a motif common to known Aβ fibril structures with two-fold or three-fold symmetry [28–30]. The presence of a structured N-terminus in this fibril is particularly interesting, because a closely related three-fold symmetric fibril topology, which was produced by *in vitro* seeding experiments, is disordered up to residue 10 [30].

The unusual three-fold symmetric geometry has gained much attention in the past and has been studied by molecular dynamics (MD) simulations regarding various aspects. The structure of the *in vitro* fibril, which was published in 2008 [30], inspired molecular modeling of different fibrillar conformers and subsequent investigation by MD simulations [33, 34]. In these studies, the disordered N-terminus was either omitted [33] or supplemented by molecular modeling techniques [34].

After the fibril structure by Lu et al. became available in 2013 [32] it served as template for some recent computational investigations. Alred et al. [35] compared the conformational stability of this fibril to that of a two-fold symmetric Aβ fibril. Other studies investigated the fibril growth mechanism by coarse grained MD simulations [36] or the strength of peptide interactions by quantum chemical methods [37]. Thus, although three-fold symmetric Aβ fibrils with structured N-terminus have already been studied by MD simulations, the structural role of the N-terminus has not yet been investigated in detail.

In the present study, we examined the particular role of the N-terminal residues for the conformational stability of an Aβ(1–40) fibril with threefold symmetry (henceforth termed “triple-fibril”). For that purpose, we performed all-atom MD simulations in explicit water both for the full-length and an N-terminally truncated fibril Aβ(9–40). In addition, the influence of the number of fibrils layers upon overall stability was addressed. We observed that fibril stacks of six or more layers remained stable for the full-length fibril, but underwent significant structural changes in the absence of the N-terminus. The type of rearrangements observed also lead us to propose a potential mechanism involved in the formation of fibrils with three-fold symmetry.

## Methods

### Preparation of starting structures

The initial simulation structures were based on the PDB entry 2M4J [32], containing three-layers of a patient-derived Aβ(1–40) fibril with three-fold symmetry. The middle layer of the first structural model was used as a template to obtain regular oligomeric structures with three, six, and twelve layers according to an established strategy [38–40]. The positions of the additional layers were constructed by adding multiples of the mean displacement vector between two adjacent layers of the PDB structure to the coordinates of the middle layer atoms. N-terminally truncated systems were obtained by substituting the first eight residues of every Aβ chain with an acetyl blocking group using SYBYL 7.3 [41]. Na^+^ counter ions were added to all systems for electrical neutralization. The oligomer structures were solvated in a TIP3P [42] water box in the form of a truncated octahedron with a minimum distance of 10 Å between the protein atoms and the borders of the box.

For the infinite fibril systems, we followed a simulation setup applied previously for an Aβ fibril with two-fold symmetry [40]. The twelve-layered Aβ oligomers were aligned with their fibril axis to the z axis, electrically neutralized by the addition of counter ions, and solvated in a cuboid water box with at least 10 Å distance between the protein and the borders. To obtain a continuous Aβ fibril system, we took advantage of the periodic boundary conditions: the outermost Aβ layers were positioned to interact with the Aβ image layers in the neighboring simulation boxes leading to an infinite fibril stack. Therefore, the distance of the outermost Aβ layers to the z edges of the simulation box was adjusted by shrinking the z dimension of the box according to the following procedure: First, the distance between the top Aβ chain in the original box and the bottom Aβ chain of the z image was measured in VMD [43]. Second, the difference between this distance and the average distance of two subsequent Aβ layers along the z axis in the template structure was determined. This difference was then subtracted from the z dimension of the box specified in the Amber restart file. Finally, water molecules outside the new box boundaries were deleted from restart file and topology file using a self-written Perl script. This setup for an infinite system utilizing periodicity appears straightforward here, since experimental studies demonstrated the absence of a twist in the present Aβ triple fibril [32].

### Molecular dynamics simulations and analysis

All simulations were performed using the Amber14 package [44] with the ff99SB force field [45, 46]. To reduce steric tensions in the initial structures energy minimizations were performed. These occurred in three steps with 5,000 optimization cycles each: First, only water molecules were relaxed keeping all other atoms restrained with a force of 10 kcal mol^-1^ Å^-2^. In the second step, Na^+^ ions and hydrogen atoms were allowed to move in addition, and finally an unrestrained minimization of the complete system was carried out. Afterwards, all systems were heated up to 310 K and equilibrated in a three-step approach following a procedure applied previously [47, 48]. Each production phase comprised 200 ns with a time step of 2 fs. Amber default settings for an NPT ensemble and the SHAKE algorithm [49, 50] were applied to constrain the hydrogen-containing bonds.

For all systems, two simulations with different initial velocity distributions were performed. While the production runs used the GPU-accelerated version of Amber [51–53], minimization and equilibration were carried out on CPUs to ensure numerical stability. An overview of all systems used in this work is given in Table 1.

**Table 1:** Overview of the Aβ systems simulated. For all systems, two simulation runs of 200 ns each were performed.

System	# Filaments	# Layers ^a^	Aβ residues	# H_2_O	# Atoms
AL_3x∞_	3	∞	1–40	24,488	95,100
AT_3x∞_	3	∞	9–40	16,503	66,897
AL_3x12_	3	12	1–40	38,302	136,542
AT_3x12_	3	12	9–40	20,053	77,547
AL_3x6_	3	6	1–40	33,395	111,003
AT_3x6_	3	6	9–40	14,903	53,403
AL_3x3_	3	3	1–40	23,577	76,140
AT_3x3_	3	3	9–40	13,137	43,758

^a^ In the infinite fibril system, the periodic box contained 12 layers; see text for details

Analysis of the resulting trajectory was performed using the AmberTools suite [54]. In order to determine the torsion angle ϕ between two filament axes, the vectors $\vec{\nu_{l}}$ between the Cα atoms of Phe19 in the two outmost filament layers were computed, the respective torsion angle between the *i*^*th*^ and *j*^*th*^ filament was calculated and averaged over all three filament pairs:

$$\varphi =\frac{1}{3}\sum_{i\neq j}^{3}\text{across}\frac{\vec{\nu_{l}}o\vec{\nu_{J}}}{\left\|\vec{\nu_{l}}\right\|\cdot \left\|\vec{\nu_{J}}\right\|}$$

Graphical representations of the molecular systems were created with VMD [43] and Chimera [55], data plots were generated by gnuplot [56].

## Results and discussion

### Global structural properties of the individual systems

**Fig 2:**
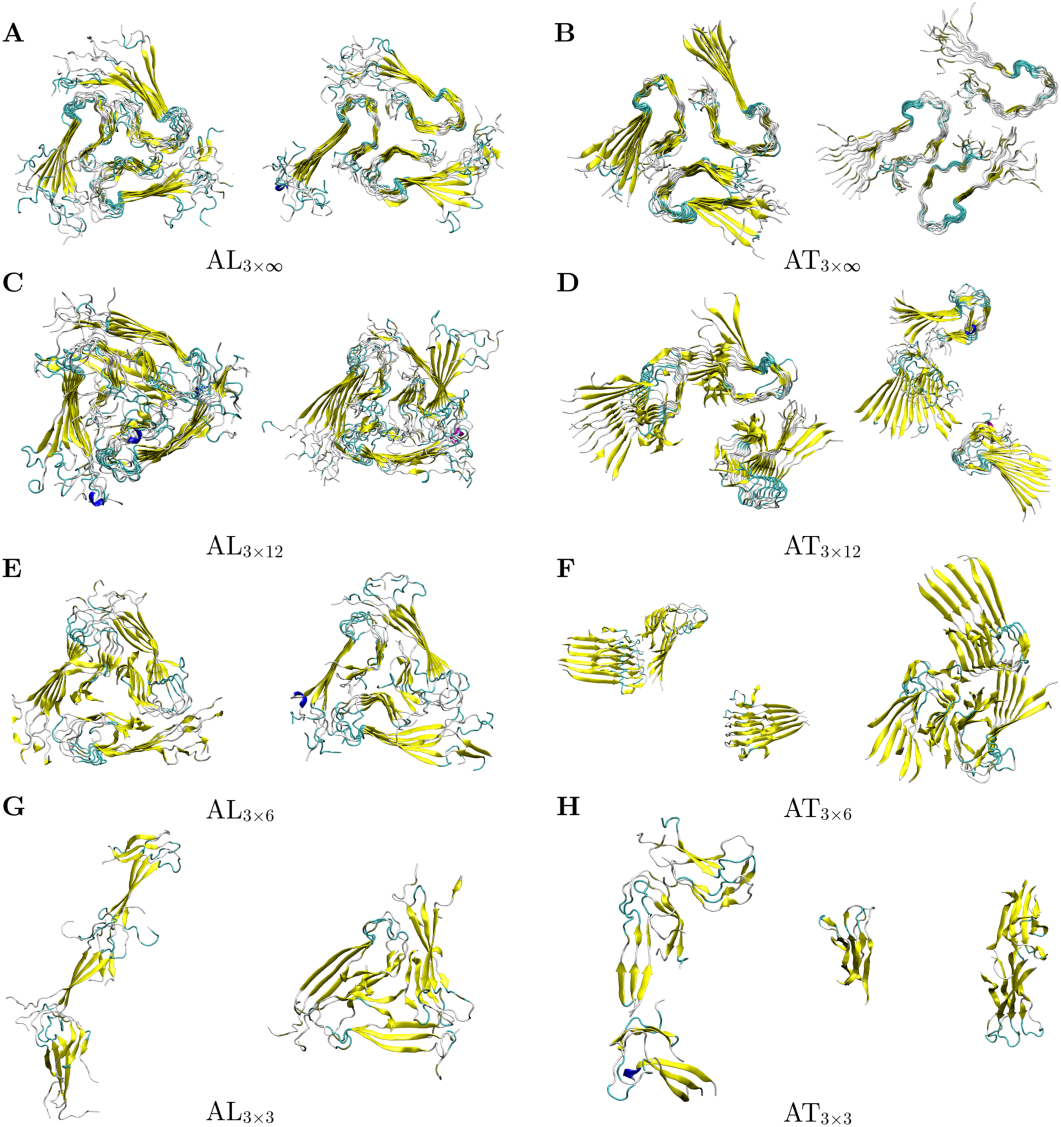
Final structures of full-length (AL, Aβ1–40) and truncated (AT, Aβ9–40) triple-fibril systems obtained from the MD simulations. The left and right panels show the structures of the two independent MD runs. Only the backbone conformation is displayed with β-sheets depicted as yellow arrows.

The present study aimed to assess the role of two structural features for the conformational stability of an Aβ40-fibril with three-fold symmetry. (i) The role of the N-terminus was assessed by simulating the fibril in its physiological full-length (residues 1–40) and in an artificially N-terminally truncated form (residues 9–40). (ii) The number of fibril layers required for the maintenance of this topology was investigated by simulating stacks of 3, 6, and 12 layers as well as an infinite stack both for the full-length (AL) and truncated (AT) form. Details of the system setup and the nomenclature used are given in Table 1. The final structures obtained for the simulation of each system are summarized in Fig 2.

The very small systems containing only three layers completely lost their initial three-fold symmetry regardless of the presence of the N-terminal residues (cf Fig 2G and 2H). The individual Aβ filaments formed a linear arrangement, showed a strong twisting of the stacks relative to each other, or underwent dissociation of one stack. Three Aβ layers are thus not sufficient to stabilize a triple-fibril conformation in accordance with previous findings [35, 36]. Thus, these systems were excluded from further analysis.

All larger full-length Aβ systems investigated, AL_3x6_, AL_3x12_, and AL_3x∞_ retained their triple-fibril conformation (cf Fig 2A, 2C and 2E), although the systems displayed a certain flexibility. The terminal Aβ chains in AL_3x6_ and AL_3x12_ lacking a second β-sheet binding partner were more mobile than the other chains, which is known from other simulation studies [38, 47, 57–59]. Overall, our findings for the full-length Aβ40 systems with respect to their structural stability are in agreement with earlier studies [35, 36].

In contrast, the triple-fibril conformation was not stable in the truncated Aβ systems AT_3x6_, AT_3x12_, and AT_3x∞_ (cf. Fig 2B, 2D and 2F). Basically, we noted two types of conformational rearrangements:

1. Dissociation of fibril stacks, as observed for both AT_3x12_ and one of the AT_3x6_ simulations. Whereas a full dissociation for one of the stacks was observed for AT_3x6_ (Fig 2F left panel), the AT_3x12_ simulations still exhibited some contacts between the individual stacks (Fig 2D). These differences can most likely be attributed to the limited simulation time of 200 ns, which does not allow monitoring the full dissociation process in the larger systems.
2. Another type of conformational rearrangement was observed in one run of the AT_3x6_ and AT_3x∞_ simulations. The respective structures adopted a 2+1 topology, in which two Aβ filaments pair along their hydrophobic C-terminal sheet, while the third Aβ filament laterally associated to that structure via its C-terminal residues (Fig 2B right panel, 2F right panel). The fact that a structural rearrangement is observed in only one of the two AT_3x∞_ simulations may be attributed to the incomplete sampling of large-scale motions on the simulated time scales.

Both dissociation and formation of a 2+1 topology show that the triple-fibril conformation is not stable without the N-terminal residues. Thus, the fibril stabilization by lateral growth known from two-fold symmetric structures [38, 58, 60] is not sufficient to compensate the missing N-terminus even in the infinite triple-fibril. Compared to its significant role for the stabilization of the triple-fibril, the role of the N-terminus for the stabilization of the individual fibril stacks appears to be rather small and full-length and truncated fibrils exhibit a rather similar content of secondary structure. Thus, we did not detect an allosteric effect of the N-terminus on the remaining parts of the peptide chain as it has been observed previously for monomeric Aβ peptides [12].

The role of the N-terminus for the stability of the triple-fibril becomes also apparent from the structural parameters compiled in Table 2. When comparing systems with the same number of fibril layers, the RMSD values are lower for the full-length compared to the truncated systems (with the exception of the triple-layered systems, which are generally instable). The mean torsion angle between the fibril axes is close to zero for the infinite AL_3x∞_ fibrils indicating that the stacks remain well aligned and that no significant twisting of the individual filaments takes place. This is in line with the experimental characterization of this triple-fibril indicating a lack of twisting [32].

**Table 2:** Global structural properties. Backbone RMSD, backbone RMSF, radius of gyration R_g_, and average torsion angle ϕ between filament axes.

System	Run	RMSD [Å] ^a^	RMSF [Å] ^a^	R_g_ [Å] ^b^	ϕ [°]
AL_3x∞_	1	5.2 ± 0.6	1.1 ± 0.6	33.3 ± 0.2	1.5 ± 0.5
	2	6.4 ± 1.4	1.7 ± 0.9	33.8 ± 0.8	1.6 ± 0.3
AT_3x∞_	1	7.5 ± 0.6	0.9 ± 0.4	30.3 ± 0.2	2.4 ± 0.5
	2	9.3 ± 1.9	2.5 ± 1.2	31.3 ± 0.6	0.8 ± 0.4
AL_3x12_	1	6.1 ± 1.1	1.7 ± 0.6	34.2 ± 0.3	7.2 ± 2.8
	2	5.9 ± 1.1	1.9 ± 0.7	34.3 ± 0.4	7.3 ± 1.3
AT_3x12_	1	14.0 ± 3.5	3.3 ± 1.1	35.4 ± 1.9	14.6 ± 4.6
	2	17.6 ± 5.1	4.8 ± 1.5	37.5 ± 3.1	17.8 ± 4.9
AL_3x6_	1	5.7 ± 1.0	1.8 ± 0.7	29.0 ± 0.2	22.6 ± 5.9
	2	5.5 ± 0.9	1.7 ± 0.7	29.0 ± 0.3	18.4 ± 4.6
AT_3x6_	1	28.9 ± 10.8	11.9 ± 4.2	41.5 ± 7.2	30.0 ±11.2
	2	12.7 ± 1.7	2.6 ± 1.0	27.9 ± 0.4	25.6 ± 5.5
AL_3x3_	1	25.0 ± 8.3	9.3 ± 2.8	37.9 ± 4.2	88.9 ± 25.5
	2	8.5 ± 1.3	2.4 ± 1.0	25.3 ± 0.6	49.3 ± 10.4
AT_3x3_	1	15.9 ± 10.7	5.5 ± 1.9	29.2 ± 2.5	60.9 ± 17.0
	2	20.9 ± 5.0	8.8 ± 3.0	34.4 ± 4.5	73.7 ± 20.5

^a^ Calculated for residues 9–40.

^b^ Averaged over all layers.

**Fig 3:**
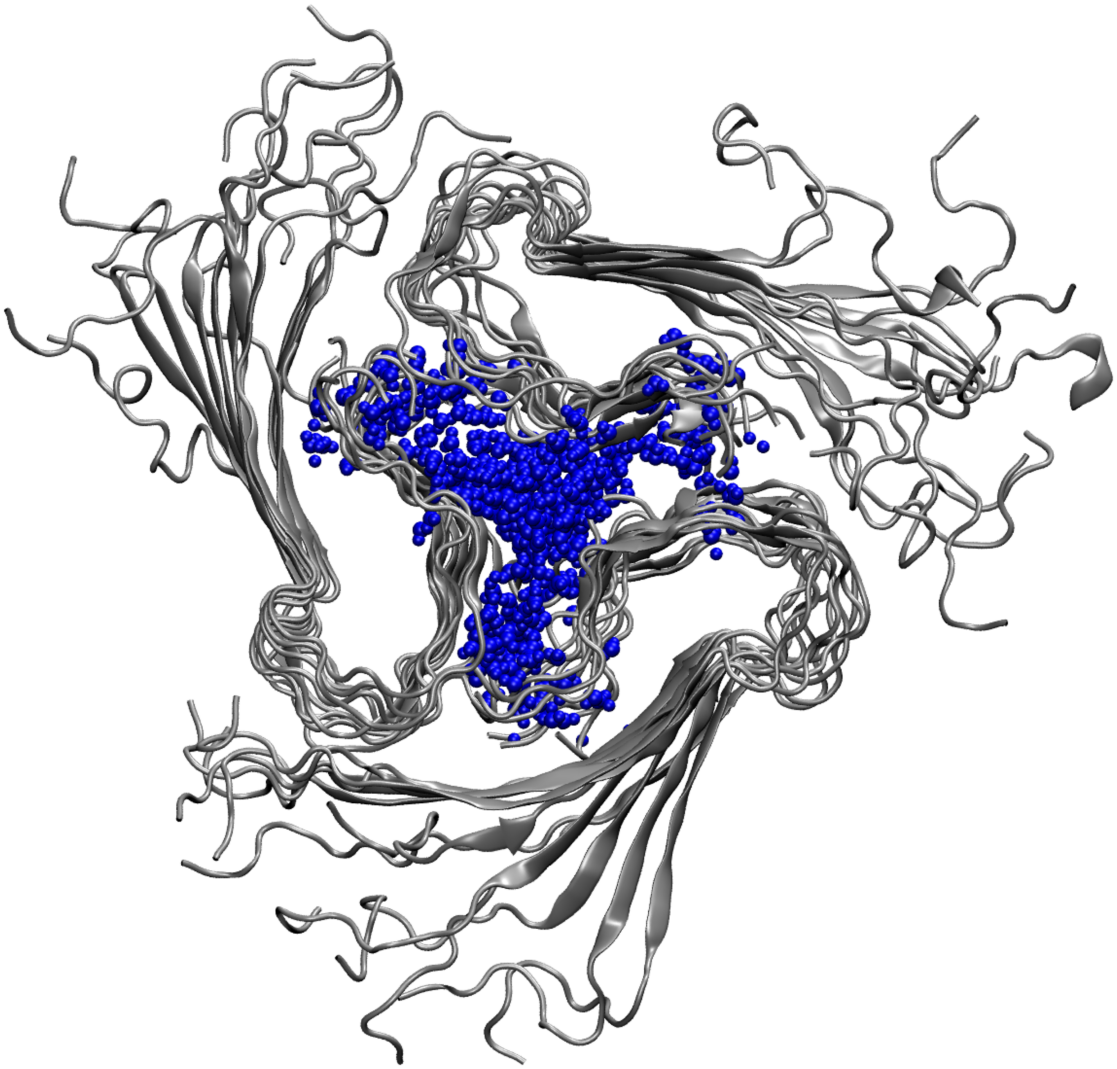
Solvation of the central water pore. The final structure of AL _3x∞_ (run 1) is shown as example. For clarity, only water molecules within a distance of 7 Å to Met35 are shown in blue.

**Fig 4:**
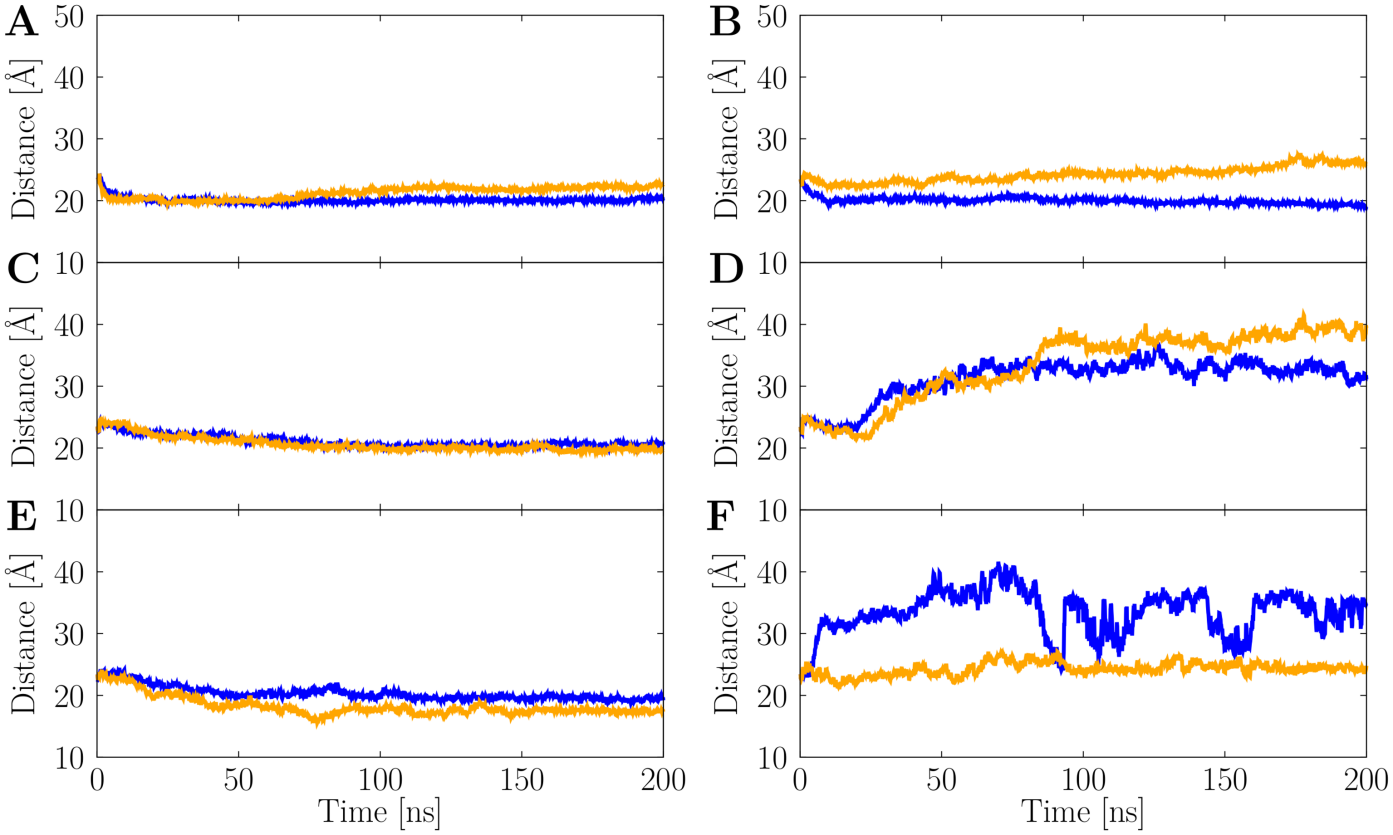
Stability of the central water pore. The average Met35-Met35 distance between Aβ chains within the same layer is shown as function of the simulation time for both full-length (left panels) and truncated (right panels) Aβ species. (A) AL_3x∞_. (B) AT_3x∞_. (C) AL_3x12_. (D) AT_3x12_. (E) AL_3x6_. (F) AT_3x6_. The two MD runs are shown in blue and orange, respectively.

The conformational differences between the full-length and truncated Aβ species may also be monitored via the stability of the central water pore, a structural feature that may relate to Aβ's cytotoxicity by disrupting membranes [61]. This water pore, which was originally described by Miller et al. [34] for triple-fibrils, is also present in our simulated structures (Fig 3). The dimensions of the pore, which is formed by the C-terminal sheets of the Aβ chains (Figs 1A and 3, can be deduced from the Met35 sidechain distances within the same layer [34, 35]. In our study, the full-length Aβ systems show a constant pore dimension of ~20 Å throughout the simulations (Fig 4), after a quick initial relaxation from the initial distance of 23 Å observed in the PDB structure. In contrary, due to structural rearrangements the truncated species AT_3x∞_ shows a drift in the distances in one of the simulations, and AT_3x6_ and AT_3x12_ water pores are instable.

In summary, our MD simulations show a clear trend for the Aβ40 triple-fibril investigated. Full length Aβ40 systems with six or more layers retain the three-fold symmetry, while the missing N-terminal residues cause a structural instability in the truncated systems resulting in dissociation or formation of a distinct 2+1 topology of the Aβ filaments. Small three-layered species on the other hand are not stable regardless of their N-terminal length.

### Stabilizing interactions of the N-terminal residues

**Fig 5:**
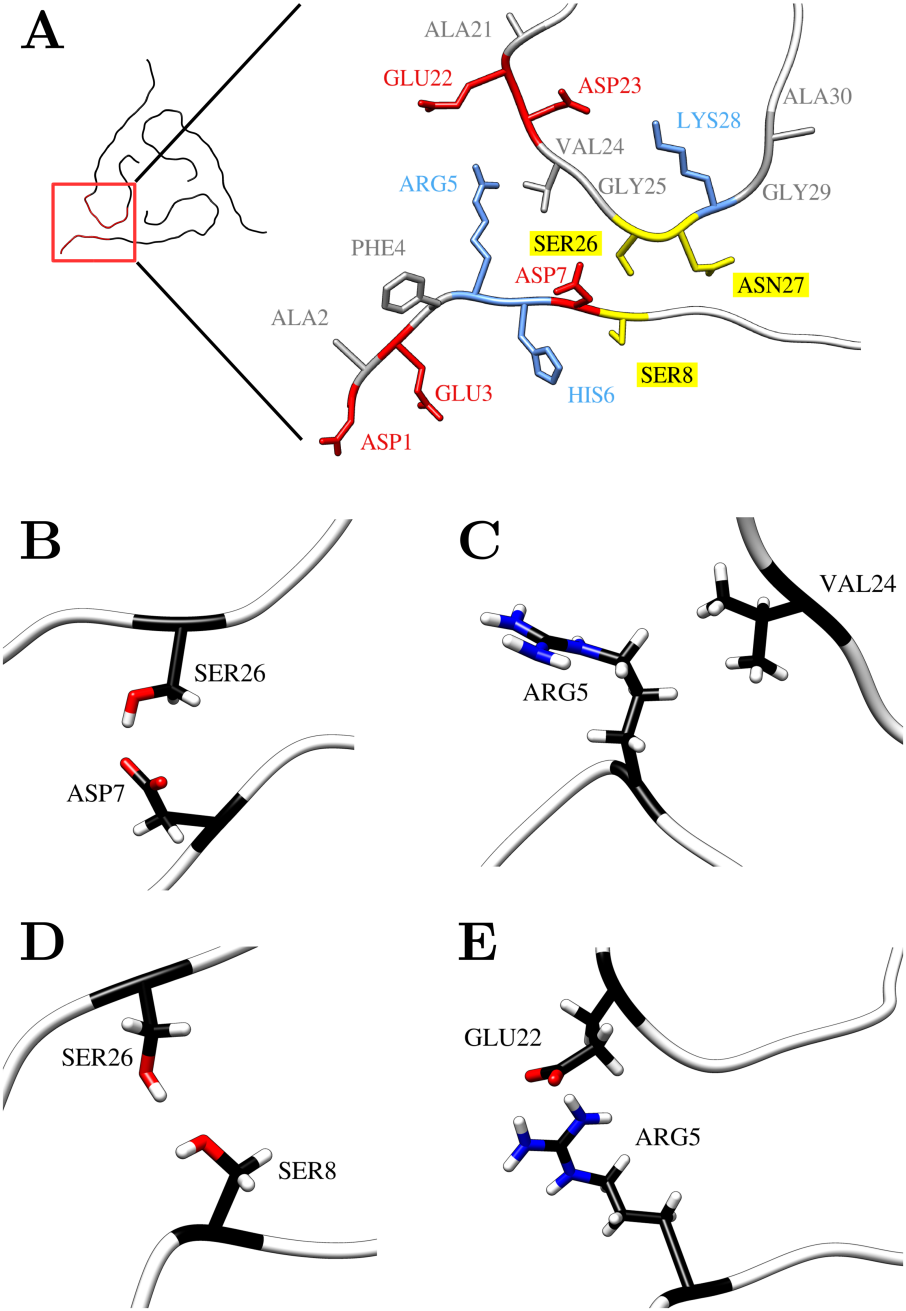
Contacts between N-terminus and the neighboring Aβ chain of the same fibril layer. (A) Orientation of the N-terminal residues 1–8 and the proximal residues 21–30 in the initial structure (hydrogen atoms are omitted for clarity). (B-E) Representative N-terminal contacts observed during the MD simulation: (B) Asp7-Ser26, (C) Arg5-Val24, (D) Ser8-Ser26, and (E) Arg5-Glu22.

Since the N-terminus clearly influences the overall structure, the role of the individual residues was analyzed in more detail. Inspection of the experimental structure suggests that the stabilizing role of the N-terminus originates from its interaction with the turn region of the neighboring Aβ chain (Fig 5A), thereby fixing the arrangement of the individual stacks. Pairs of interacting polar residues include Ser8-Ser26, Asp7-Ser26, and Arg5-Glu22. Furthermore, hydrophobic contacts are formed between the alkyl chains of Arg5 and Val24. However, the geometry was not optimal for most of these interactions in the initial structure. Therefore, it was of particular interest to investigate, whether these interactions remain stable during the MD simulation and also whether additional interactions are formed.

**Fig 6:**
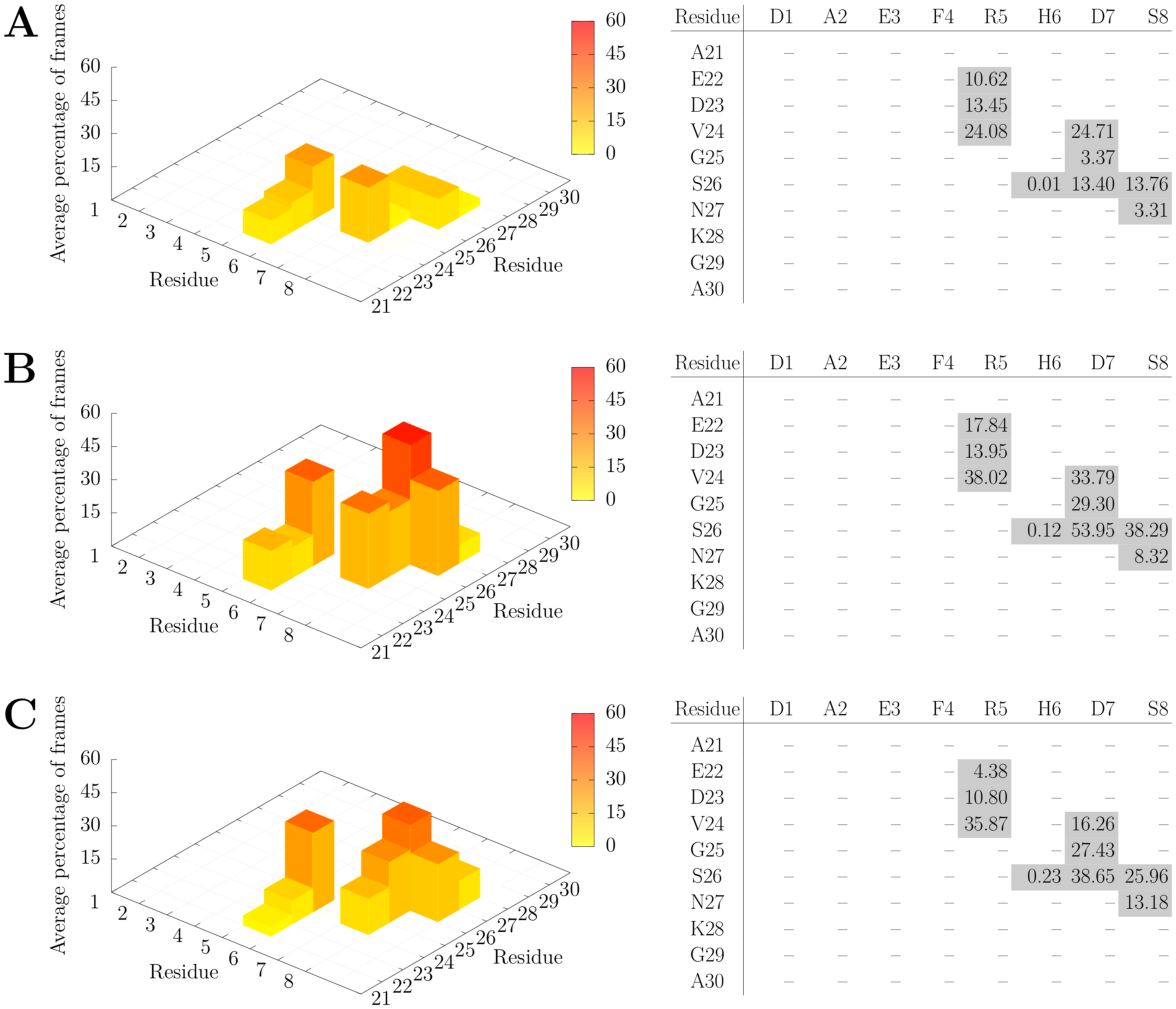
Atom-atom contacts (distance cutoff 4 Å) between the N-terminal residues 1–8 and the neighboring residues 21–30 of the next Aβ chain for AL_3x∞_ (A), AL_3x12_ (B), and AL_3x6_ (C). Numbers are mean percentage values over all layers and MD runs. See Fig 5A for a structural representation of the two sets of residues.

To cover all potential contacts originating from the N-terminus, the interactions between turn residues 21 to 30 and N-terminal residues 1–8 were analyzed in detail. Fig 6 shows the average frequency of contacts between the two stretches of residues in fibrils with different number of layers.

First of all, there is a striking similarity in the interaction pattern in all systems independent from the number of layers. Of the eight N-terminal residues, only Arg5, Asp7, and Ser8 establish contacts with a significant frequency. All contact pairs deduced from the experimental structure, i.e. Arg5-Glu22, Arg5-Val24, Asp7-Ser26, and Ser8-Ser26, were found in the MD simulation. Additionally, there arose contacts between Arg5 and Asp23, Asp7 and Val24, as well as between Ser8 and Asn27 (Fig 6). Structural examples for the major contacts listed in Fig 6 are presented in Fig 5B and 5E. The different types of interactions include hydrogen bonds Asp7-Ser26 and Ser8-Ser26, the salt-bridge bridge Arg5-Glu22, and the nonpolar interaction Arg5-Val24.

**Fig 7:**
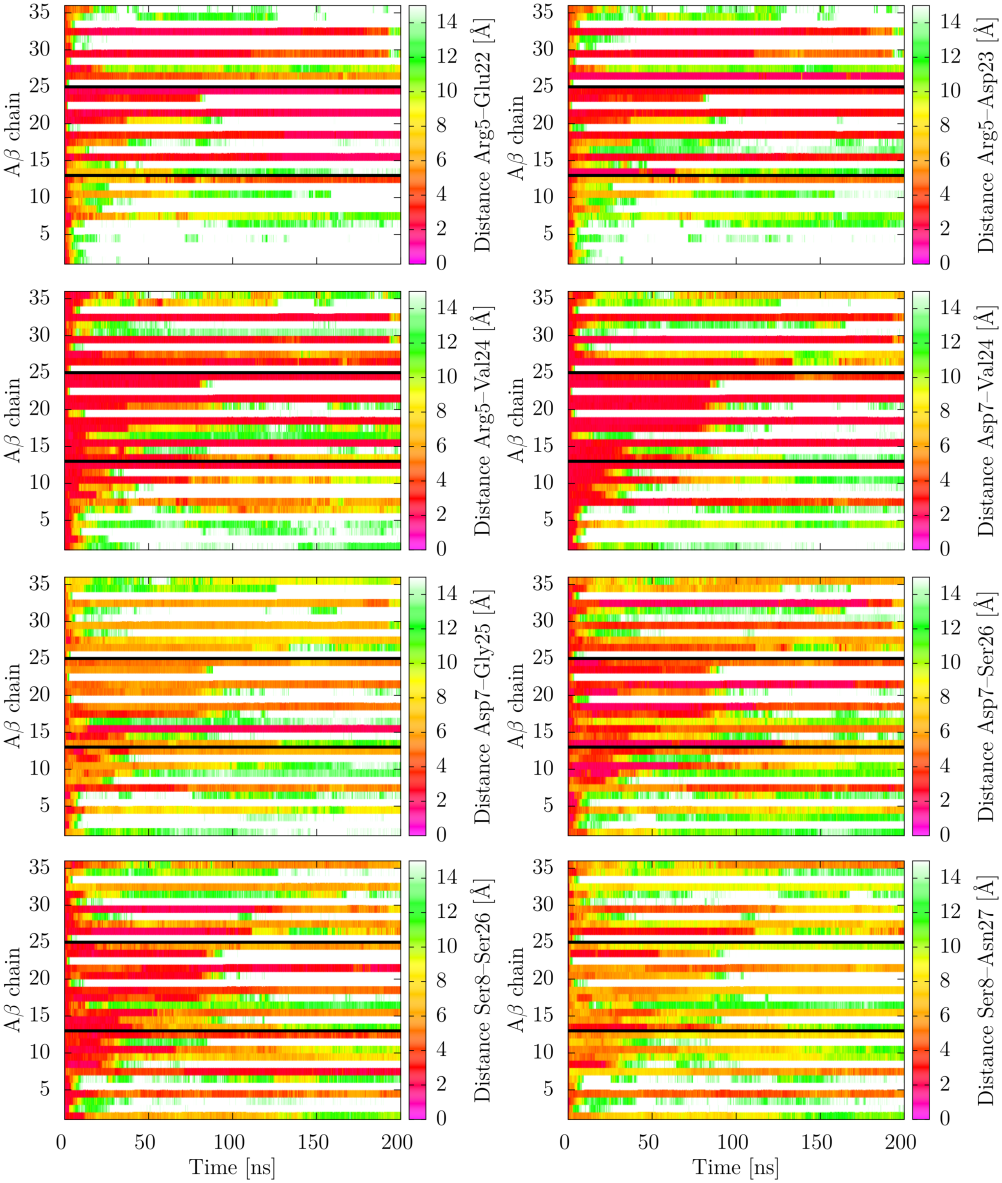
Time course of selected distances between N-terminal and core residues for each Aβ chain in the AL_3×∞_ (run1) fibril. The distance analyzed is given as right label of each panel. The y axis shows the respective interacting Aβ chains: From bottom to top, interactions of filaments 1↔2, 2↔3, and 3↔1 are presented, separated by black lines. The chains are in the same order as in the fibril stack (i.e. line 1 shows the 1↔2 in the first fibril layer, whereas line 36 shows the 3↔1 in the 12^th^ fibril layer).

**Fig 8:**
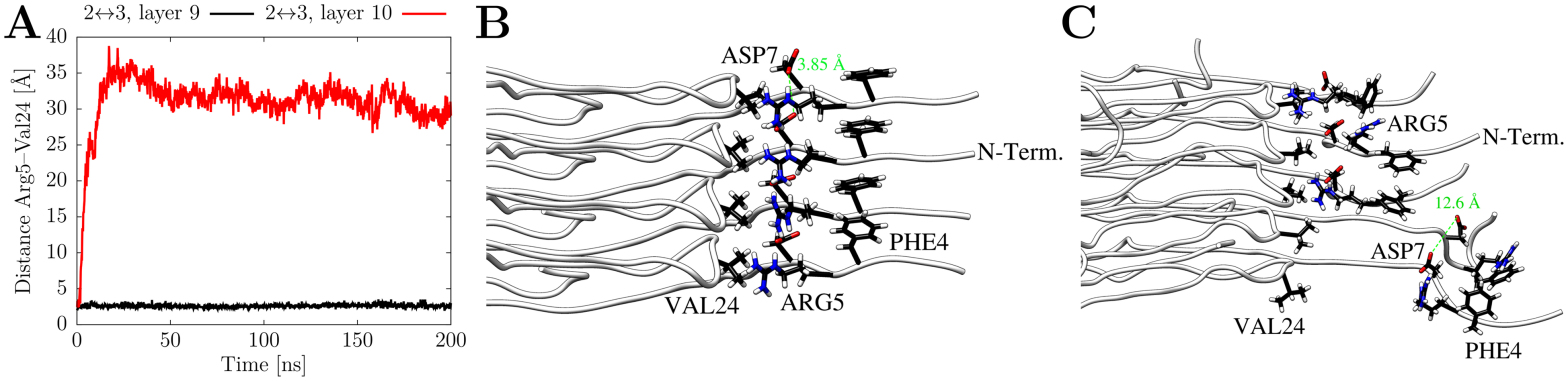
Details of the contacts between N-terminus and loop region in AL_3×∞_ (run1). (A) Representative plot of contact distances Arg5-Val24 for two adjacent layers as a function of simulation time. (B) Initial structure with dense packing of residues Arg5, Asp7, and Phe4 in adjacent layers; the distance between two Asp7 residues in adjacent chains is 3.85 Å. (C) Representative snapshot structure with the Asp7-Asp7 distance increased to 12.6 Å.

In order to have a closer look into the dynamical behavior of the interactions formed by the N-terminal residues, we analyzed the evolution of the respective distances over the simulation time. This more detailed analysis allowed dissecting the average values shown in Fig 6 and revealed that the rather low percentage of contacts detected in Fig 6 rather result from differences between the individual layers than from large fluctuations over time within each layer. This behavior was generally observed in all simulations of the full-length triple fibril and is shown for run 1 of AL_3x∞_ in Fig 7. Fig 8A exemplarily depicts this different behavior for the contact Arg5-Val24 in the 9^th^ an 10^th^ layer of AL_3x∞_ showing that the contact remains stable in layer 9 whereas it is rapidly lost in layer 10. A possible explanation for this alternation might be the system's conformational response to the electrostatic repulsion of the charged residues Arg5 and Asp7 due to the parallel in-register orientation of the Aβ chains within a single filament: The location in the terminus allows for a larger local flexibility of the individual peptide termini without compromising the interactions of adjacent termini with the core (cf Fig 2A, 2C and 2E). The spatial arrangement of these charged amino acids, which potentially leads to the alternating interaction pattern, is depicted in Fig 8B and 8C. The distance between two Asp7 residues in neighboring chains changes from 3.8 Å in the initial structure to 12.6 Å in one of the layers that undergo conformational rearrangement during the simulation.

In both conformations shown in Fig 8B and 8C, Phe4 forms stacking interactions between adjacent layers thus stabilizing the fibril stack. This mode of stabilization is slightly different from a previous work, in which interactions between Phe4 and Gly25 were observed [34]. These differences can most likely be attributed to the model building procedure, in which Phe4 was either modeled to interact with Gly25 [34] or to form Phe4-Phe4 stacking interactions according to the structure by Lu et al.[32].

In summary, the data presented in Figs 6 and 7 shows that there is some plasticity in the interactions formed by residues Arg5, Asp7, and Ser8 of the N-terminus. Notably, only these three polar residues are involved in fixing the conformation of the N-terminus in the fibril investigated in the present study.

Our analyses thus show that the N-terminal residues play a pivotal role in the stabilization of the Aβ40 triple-fibril structure. In their absence, the interaction between the single filaments is too weak to preserve the three-fold symmetry.

### Characterization of the 2+1 topology and a suggested mechanism involved in triple-fibril formation

In two simulations of the N-terminally truncated triple-fibrils, a conformational rearrangement to a 2+1 topology was observed. In this topology two Aβ filaments paired along their hydrophobic C-terminal sheet, while the third Aβ filament remained laterally associated to that structure via its C-terminal residues.

**Fig 9:**
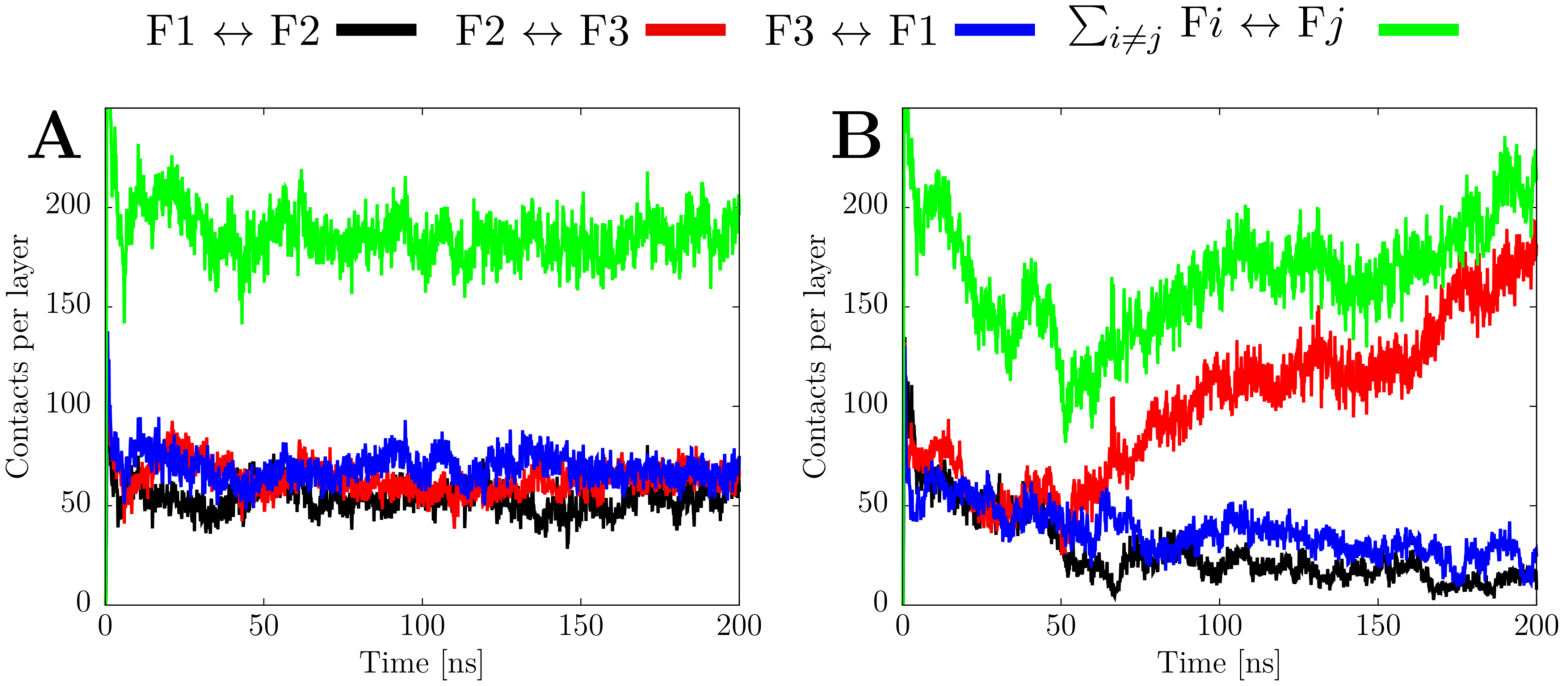
Interfilament contacts in AT_3x∞_ for run1 (A) and run2 (B). The atom-pair contacts between two of the filaments are depicted in black, red, and blue, respectively (cutoff: 4 Å); the sum of all inter-filament contacts is shown in green.

To characterize the mechanism of this conformational change, we investigated the AT_3x∞_ system in more detail. Fig 9 shows the evolution of the contacts between the individual filaments over the course of both AT_3x∞_ simulations. In the first MD run, in which the original triple-fibril structure is retained (Fig 2B left panel), the number of ~ 60 interfilament contacts is similar for all three interfaces, yielding a total number of ~ 180 contacts per layer within the triple-fibril (Fig 9A). In the second run, where the 2+1 topology formed (Fig 2B right panel), the situation is different. While the contacts between filament F1 and the two others (F2, F3) decrease over the simulation and reach a value of ~ 20 at the end, the interface between F2 and F3 becomes reinforced after 50 ns and the number of contacts increases to ~ 160 (Fig 9B). The number of total contacts first decreases thus reflecting the initial destabilization of the triple-fibril topology. After 50 ns, the gain of interactions between filaments F2 and F3 also leads to an increase of the total number of contacts (Fig 9B). This behavior, in which the contacts drop at first and then rise again, reflects the refolding process from the triple-fibril to the double fibril with a loosely attached third filament. Notably, the total number of contacts at the end of the simulation is rather similar for both AT_3x∞_ indicating that the 2+1 topology represents an alternative stable conformation to the triple-fibril topology (Fig 9).

**Fig 10:**
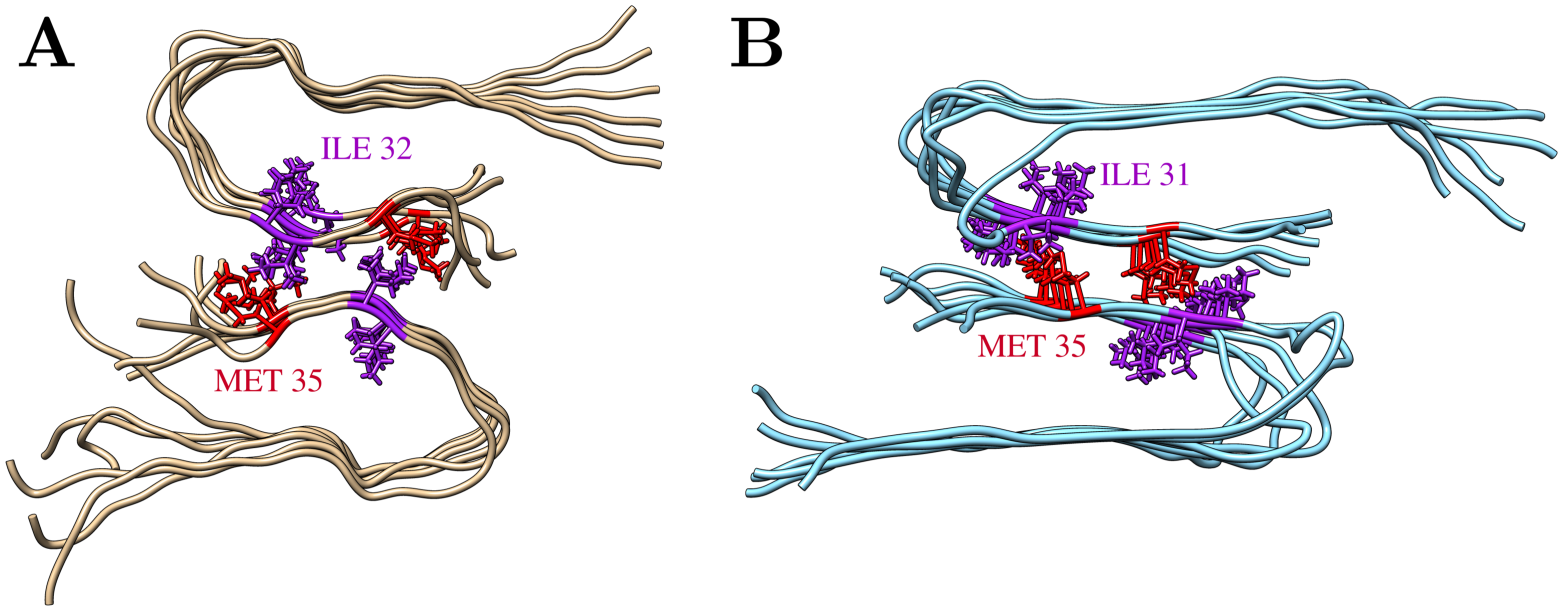
Comparison of the C-terminal interface between two filaments. (A) Final structure of the AT_3x∞_ simulation (run2; the third loosely attached filament is omitted for clarity). (B) Twofold symmetric Aβ40 fibril structure determined by solid-state NMR spectroscopy (PDB 2LMN [30]). Interacting residues (Met35, Ile31/32) are shown in sticks.

One hallmark of the 2+1 topology is the tight interaction between two of the filaments via their C-terminal strands (Fig 10A). Interestingly, this mode of interaction shares some resemblance to the experimentally observed interface in fibrils with twofold-symmetry (Fig 10B)

In both arrangements, the C-terminal residues associate in an antiparallel fashion and Met35 is a key residue of the interface. However, the interaction partner of Met35 differs between the two interfaces. In the large hydrophobic interface of the 2+1 topology Met35 interacts with Ile32, whereas it contacts Ile31 in the two-fold symmetric fibril (Fig 10). This different inside-outside topology around Ile31/Ile32 is not due to a conformational rearrangement during MD simulations, but is already present in the experimentally determined structures of the triple- and double-fibril. In the triple-fibril, the C-terminal residues deviate from a β-strand topology at Gly33 thus allowing Met35 and Ile32 to point to the same side. This property is in line with the large plasticity of β-sheet topology, which tolerates various inside-outside topologies in Aβ fibrils [62].

Our MD simulations reveal only minor conformational changes within the filaments upon formation of the 2+1 topology including a more extended conformation for Ile32/Gly33 peptide bond and a C-terminal extension of the β-strand to Val36. This finding suggests that both triple and 2+1 conformation can be readily interconverted. This is in line with experimental data showing that alternating regions with two and three parallel filaments within a single fibril may occur [63].

**Fig 11:**
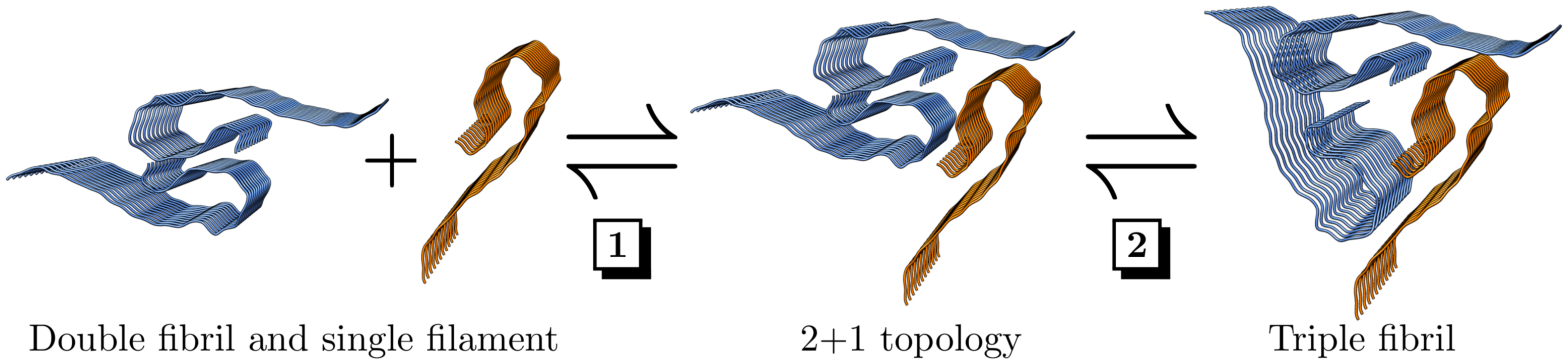
Suggested two-step mechanism for the formation of Aβ triple fibrils. In the first step (**1**), a double-layered fibril binds a third single filament forming a 2+1 topology. In the second step (**2**), the fibril converts into a symmetric three-fold symmetric structure.

The fact that we have observed this 2+1 topology only for the N-terminally truncated but not for the full-length Aβ systems may be explained by the following consideration: The triple-fibril, but not the 2+1 topology relies on interactions of the N-terminus for its stabilization. Thus, an N-terminal truncation as performed in our simulations mainly destabilizes this triple-fibril conformation. This in turn allows monitoring filament dissociation and formation of the 2+1 topology on the timescale of our MD simulations. For the full-length filaments the conformational equilibrium is expected to be shifted towards the triple-fibril due to the stabilizing interactions of the N-terminus. The considerations above prompted us to propose a potential two-step mechanism involved in the formation of triple-fibrils from double fibrils by incorporation of a single filament (Fig 11). In the first step, an existing double-fibril binds an isolated filament. In the second step, the resulting aggregate undergoes a conformational rearrangement to a symmetric triple-fibril topology. However, the conformational equilibrium of different topologies may strongly be influenced by the peptide sequence as well as the surrounding environment and more comprehensive thermodynamic analyses will be required to finally prove the mechanism of formation for this and maybe even other triple-fibril topologies [30, 33, 64].

## Conclusions

Our study suggests that the relative stability of different Aβ fibril topologies may depend on the properties of the N-terminus. Since the key residues Arg5, Asp7, and Ser8 necessary for stabilizing interactions of the N-terminus are polar, perturbations affecting electrostatic interactions are expected to have a significant impact on the overall fibril topology observed. Such perturbations may include external factors like pH value or ionic strength, but also internal factors, like posttranslational modifications, mutations that affect charge/polarity (e.g. English His6Arg and Tottori Asp7His mutant) or N-terminal truncations. Environmental factors in turn are likely a reason for the fact that unique fibril structures were isolated from different patients [32].

Thus our study adds evidence that the N-terminal residues may not only play a role in the initial steps of fibrillation [27], but also at least in part be responsible for the large variety of fibril polymorphisms [65] observed. We conclude that the N-terminal Aβ residues significantly influence the amount of Aβ fibril polymorphism in an organism or tissue [66] in interplay with other physiological factors in the cell like lipid bilayers, molecular chaperones, and macromolecular crowding.
