# Correction: Role of the N-terminus for the stability of an amyloid-β fibril with three-fold symmetry

**DOI:** 10.1371/journal.pone.0189238

**Published:** 2017-12-04

**Authors:** The PLOS ONE Staff

There are errors in the third paragraph under the subheading "Molecular dynamics simulations and analysis" in the Methods section. The publisher apologizes for the errors. Please view the complete, correct paragraph and equation here:

Analysis of the resulting trajectory was performed using the AmberTools suite [54]. In order to determine the torsion angle ϕ between two filament axes, the vectors ${\vec{v}}_{i}$ between the Cα atoms of Phe19 in the two outmost filament layers were computed, the respective torsion angle between the *i*^*th*^ and *j*^*th*^ filament was calculated and averaged over all three filament pairs:

$$\phi =\frac{1}{3}\sum_{i\neq j}^{3}\arccos\frac{{\vec{v}}_{i}o{\vec{v}}_{j}}{\left\|{\vec{v}}_{i}\right\|\cdot \left\|{\vec{v}}_{j}\right\|}$$
